# Famine and disease in Nigerian refugee camps for internally displaced peoples: a sad reflection of our times

**DOI:** 10.1093/qjmed/hcw171

**Published:** 2016-10-18

**Authors:** S. D. Taylor-Robinson, O. Oleribe

**Affiliations:** From the 1 Division of Digestive Health, Department of Surgery and Cancer, 10th Floor QEQM Wing, St Mary’s Hospital Campus, Imperial College London W2 1NY, UK,; 2 Excellence and Friends Management Care Centre (EFMC), Dutse Abuja FCT, Nigeria,; 3 Royal College of Physicians of London, 11 St Andrews Place, Regent’s Park, London NW1 4LE, UK and; 4 West African College of Physicians (WACP), 6 Taylor Drive, Off Edmond Crescent, Yaba Lagos, Nigeria Address correspondence to: Prof Simon D. Taylor-Robinson, Division of Digestive Health, Department of Surgery and Cancer 10th Floor QEQM Wing, St Mary’s Hospital Campus, Imperial College London, South Wharf Street, London W2 1NY, UK. email: s.taylor-robinson@imperial.ac.uk

## Background

**Figure 1:**
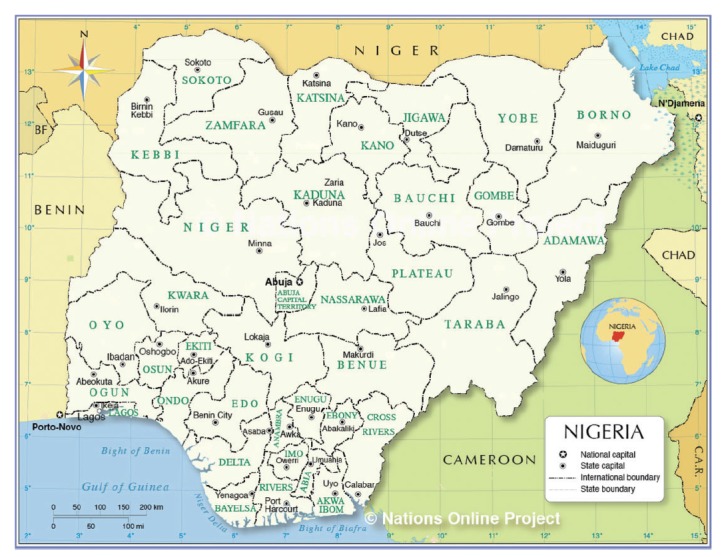
Map of Nigeria. Reproduced with permission.

Nigeria is a land of great paradox. It is Africa’s most populous nation, an oil-rich land with significant gold, iron ore, tin, uranium and coal reserves, but with large sections of the over 181 million population left in grinding poverty (Central Intelligence Agency, 2016).^1^ The country has a complex ethno-sociological mix of peoples which echo an extremely varied land that stretches from the edge of the Sahara through the savannas of the Sahel and the vast, high altitude Jos plateau to tropical rain forests, steamy coastlines and river deltas (Figure 1—map of Nigeria).^2^ A physically beautiful land with deeply hospitable people, Nigeria faces an uncertain future with segments of the country currently seeking self-determination, such as in the Niger Delta and at the risk of repeating past conflicts, the Igbo peoples in the south-east of Nigeria.^3^

Today, Nigeria lies across a fault line in Africa between the Muslim north, where Sharia law is commonplace, and the largely Christian south. This religious divide has added to the strong tribal identities which first became apparent to the outside world when the mainly Igbo south-east of Nigeria attempted to secede from the Federal Republic, declaring an independent Biafran Republic which lasted nearly three years from 1967 to 1970.^4^ The bloody war that ensued between the separatist Igbo regime in Enugu and the Federal Nigerian government, saw up to 3 million civilian deaths, mainly from starvation and the effects of chronic malnutrition, in a country that was only beginning to stand on its own feet, following independence from the UK in 1960. The bitter wounds of the Biafran war have ostensibly healed, but dig under the surface and memories are still fresh in those who survived.

In the mind of an outsider, Nigeria conjures up pictures of a weak central government, widespread corruption and inefficiency in delivery of healthcare to those in need.^5^ More recently, the Boko Haram group which preaches a local, radical form of Islam has strayed beyond the north-east of the country to inspire an insurgency against the Federal government.^6^ Bombings and targeted military-style attacks on police stations, churches and schools have been frequent and it is not surprising that large numbers of people have fled areas of conflict for those of comparative safety, including around the Federal Capital, Abuja. Although the current government have claimed some success in beating back Boko Haram to the far north-east, the internally displaced peoples face food shortages on the scale last seen in the Biafran war of the 1960s. The Internal Displacement Monitoring Centre estimates that there are almost 2 200 000 internally displaced people in Nigeria as of 31 December 2015.^7^ The same report claims that there are about 13 500 such refugees in Abuja, the Federal Capital Territory. In Abuja, a town that is wealthy by any African standard and pretty prosperous by European standards, there are a number of camps where the internally displaced are segregated and kept.

**Figure 2:**
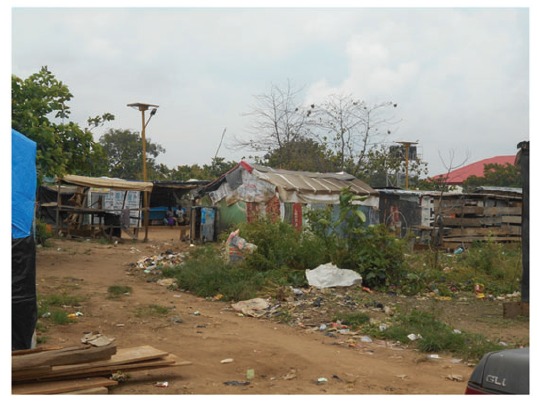
The Kuchigoro Camp in Abuja, Nigeria.

**Figure 3:**
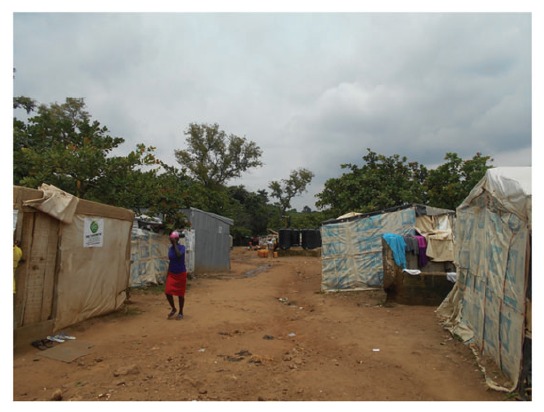
The main street in the Kuchigoro Refugee Camp.

The Kuchigoro Camp is one the refugee camps for internally displaced people from the Boko Haram insurgency. In the shadow and under the nose of the government lies a makeshift township which has sprung up with kwashiorkor and marasmus evident in the small children, no running water or easy clean water supply, a total lack of common amenities and no real evidence of governmental support (Figure 2). Apart from allowing displaced people to occupy the land, the local and federal governments have done little to improve the daily lives of internally displaced refugees on their doorstep. This is despite that fact that Kuchingoro camp is close to plush government offices, six-lane highways, smart residential neighbourhoods and expensive shops (Figure 3). Add to this the diseases of the poor, tuberculosis, malaria, measles, sexually transmitted diseases (STDs) including HIV, and childhood diarrhoea and the outlook is pretty grim. As earning money from mainstream sources is almost impossible, many take to the illicit selling and the use of hard drugs, with prostitution by non-brothel based females who sell sex, accompanied by the inherent risks of HIV, hepatitis B and other STDs. As there are a few non-governmental organizations supporting these camps, the government is seemingly blinded to the situation and little attention has been drawn to the problem in the outside world.

## What to do about the problem?

Recently, the Government of Nigeria has, in a sweeping move to improve efficiency, appointed new heads to five fundamental public health institutions including the Nigerian Centre for Disease Control (NCDC), the National Agency for the Control of AIDS (NACA), the National Primary Health Care Development Agency (NPHCDA), the Nigerian Institute of Medical Research (NIMR) and the National Health Insurance Scheme (NHIS).^8^^,^^9^ Apart from the National Agency for Food and Drug Administration and Control with an acting Director, these are some of the most important agencies in the Nigerian health sector.

Although the Nigerian Institute for Medical Research (NIMR), which oversees all aspects of research in the health sector, has not done well over the years and cannot compare favourably with sister organisations in other African countries, it is expected that with this change in leadership, NIMR will reposition to begin to make a difference in Nigeria. Assessing the impact of Boko Haram on the health of the afflicted, while measuring the prevalence of communicable and non-communicable diseases including post-traumatic stress disorder, will be pressing challenges for the new Director General to address.

The National Agency for the Control of AIDS, which coordinates the response to HIV/AIDS/STD programmes in Nigeria, was in the news recently over poor management of Global Fund grants to Nigeria.^10^^,^^11^ Providing comprehensive HIV services for the people living in these camps to ensure no new infection, to limit HIV-related deaths and to stimulate an atmosphere where there is minimal or no stigmatization may be an excellent way to renew the public trust in this very important Agency of the Federal Government.

As at the time of this report, there have been no basic healthcare services in these refugee camps. The NPHCDA, which leads healthcare policy formulation for the primary health care system, guides the delivery of basic health care services to all Nigerians at the primary care level. The NPHCDA should be able to make the establishment of basic healthcare facilities and the provision of quality healthcare services a priority at this season of change in governmental strategy. In addition, the proposed centre(s) should also provide clean drinking water, mosquito nets and environmental sanitation services to ensure that inhabitants of these camps have a meaningful quality of life.

To ensure access to essential healthcare services (beyond the primary care level), the NHIS with the mandate of ensuring that every Nigerian has access to good health services, should put mechanisms in place to establish community-based health insurance schemes (CBHIS) for the refugee camps, enrol all women and children and give them access to quality health care.^12^ CBHIS has the potential to protect internally displaced families from the financial hardship of massive medical bills, catastrophic health expenditures resulting from out-of-pocket payments, while limiting the rise in the cost of healthcare and maintaining higher standards of healthcare delivery.

Finally, the NCDC, which is charged primarily with preventing and controlling communicable diseases in Nigeria and coordinating the public health response to and preparedness for disease outbreaks, should ensure that outbreaks are prevented in these camps; and that ongoing ones are nipped on the bud as quickly as possible. NCDC is also in a position to monitor the epidemiology of diseases in these camps and should use their findings to guide policy decisions at the federal government level.

## Outside aid

While calling on the Federal Ministry of Health and all the associated Departments and Agencies to ensure that healthcare services are provided and health emergencies are prevented in these camps, the outside world should not leave the entire work to Nigeria alone. The World Health Organization, ECOWAS and the Commonwealth of Nations should partner with Nigeria to ensure lives are saved, communities restored and refugee camps are emptied as internally displaced people are supported to return to their communities and villages. Also, lessons learned from these camps should be used to ensure future emergencies are better handled with minimal morbidity and mortality.

## Role of NGOs

An example of a NGO active in this area in Nigeria is the Excellence and Friends Management Care Centre (EFMC), based in Abuja, which is a not-for-profit, public health service oriented organization committed to human development, system re-engineering and disease containment.^13^ It works to improve the lives of men and women using effective, efficient, locally generation solutions in lasting ways. Through rationalization or ‘commonization’, integration, and decentralization of public health services, EFMC works to reach those in need, particularly in marginalised sections of society.^14^ EFMC has staff with expertise in diverse areas of public health, including health services, leadership and management, education and training, nutrition and environmental resources, civil society and human/child rights, gender equality and equity, research and technology. For Nigeria, these resources make EFMC an uncommon one-stop centre for many public health solutions, as the organisation has the capacity to address some of Nigeria’s interrelated development challenges.

EFMC supports the Federal Government of Nigeria and all other key stakeholders to “communize” HIV services in Nigeria for the benefit of all, providing services in hard to reach areas of the country, while ensuring full integration and diversification of care, and enhancing sustained cross-sectional integration of HIV/AIDS services.^15^^,^^16^ EFMC also provides training, mentoring and capacity development for health workers, empowering them to provide and deliver sustainable high quality, comprehensive, prevention, treatment, care and related services in most states of Nigeria and beyond. Over the past 5 years, EFMC has provided HIV Testing and Counselling services to over 600 000 people, including to 160 000 pregnant women, with highly active anti-retroviral therapy administered to more than 7500 people living with HIV, care and support to over 11 000, including more than 4000 orphans and vulnerable children.

With a head office in Abuja, EFMC can partner with the Nigerian Federal Ministry of Health and any of its agencies to improve the quality of life of internally displaced people in refugee camps in Abuja. EFMC would be able to work with NCDC to investigate and control disease outbreaks, with NHIS to establish a community health-insurance scheme, and with NPHCDA to provide primary healthcare services for the camps. Also, working with NACA, EFMC can identify individuals infected with HIV, provide comprehensive care and prevent new infections within and around refugee camps. Recent EFMC activity in these Abuja refugee camps has highlighted dire need, but with the right support and funding, EFMC can partner with the governmental agencies to give the camps a new face.

## Conclusion

Although the plight of the internally-displace peoples of Nigeria is currently a major blight in this part of West Africa, efforts at service reorganisation by the Nigerian Federal Government, together with the efforts of NGOs and the international community still have the potential to ensure that current problems do not become another Biafran conflict, which wounded Nigeria for so long.
